# Efficacy and safety of selective internal radiation therapy with yttrium-90 for the treatment of unresectable hepatocellular carcinoma

**DOI:** 10.1186/s12876-021-01805-6

**Published:** 2021-05-12

**Authors:** Nguyen Van Thai, Nguyen Tien Thinh, Thai Doan Ky, Mai Hong Bang, Dinh Truong Giang, Le Ngoc Ha, Mai Hong Son, Dao Duc Tien, Hyun Woong Lee

**Affiliations:** 1 Department of Hepato-Gastroenterology, 108 Military Central Hospital, 1 Tran Hung Dao Street, Hai Ba Trung District, Hanoi, Vietnam; 2 Department of Nuclear Medicine, 108 Military Central Hospital, Hanoi, Vietnam; 3 Department of Hepato-Gastroenterology, 175 Military Hospital, Ho Chi Minh, Vietnam; 4 Department of Internal Medicine, Gangnam Severance Hospital, Yonsei University College of Medicine, 211 Eonju-ro, Gangnam-gu, Seoul, 06273 Korea

**Keywords:** Hepatocellular carcinoma, Selective internal radiation therapy, Yttrium-90, Survival, Tumor response

## Abstract

**Background:**

This retrospective analysis was undertaken to evaluate the efficiency of SIRT with Y-90 microspheres and determined prognostic factors affecting patients with unresectable HCC.

**Methods:**

A total of 97 patients diagnosed with unresectable HCC who underwent SIRT with Y-90 microspheres. Patient survival was assessed using the Kaplan–Meier method, and prognostic factors affecting survival were assessed using log-rank tests and Cox proportional hazards regression.

**Results:**

Among the 97 patients (90 males, mean age 60.4 ± 12.3 years) who underwent SIRT, the median clinical follow-up was 16.4 (1.8–62) months. The median overall survival (OS) was 23.9 ± 2.4 months. Tumor response according to the Modified RECIST in patients followed up beyond 6 months included a complete response (CR) to treatment in 12 patients (18.8%), partial response (PR) in 23 (35.8%), stable disease (SD) in 8 (12.5%), and progressive disease (PD) in 21 (32.8%). Factors associated with longer OS included age > 65 years, BCLC stage B, tumor size < 5 cm, tumor burden < 25%, and tumor response (CR/PR). In multivariate analysis, unilobar disease and objective tumor response (CR/PR) were predictors of longer OS.

**Conclusion:**

SIRT was an effective treatment for unresectable HCC. Unilobar disease before SIRT and tumor response (CR/PR) were positive prognostic factors.

## Introduction

Hepatocellular carcinoma (HCC) is the most common primary liver cancer. Worldwide, liver cancers are the fourth most common cause of cancer-related deaths. Based on annual projections, the World Health Organization estimates that more than 1 million patients will die from liver cancer in 2030. In the United States, the proportion of deaths owing to liver cancer increased by 43%, from 7.2 to 10.3 deaths per 100,000, between 2000 and 2016, with a 5-year survival rate of 18% [1]. However, only 20–30% of HCC patients are diagnosed at an early stage; most (> 70%) patients are diagnosed with unresectable disease and have poor overall prognosis [2]. Radical therapies, including resection or transplantation, are the gold standard for early-stage HCC [3]. Locoregional therapies such as transarterial embolotherapies, including conventional transarterial chemoembolization (TACE), bland transarterial embolization, and drug-eluting bead TACE (DEB-TACE) have played an increasingly important role for patients with unresectable HCC. Targeted molecular therapies also have a recognized role, with sorafenib (regorafenib and lenvatinib) showing improved survival in advanced HCC patients [4]. SIRT with yttrium-90 (Y-90) microspheres is another feasible treatment option for this patient group, with a disease control rate of approximately 80% [5]. SIRT is usually indicated for intermediate- or advanced-stage patients who are poor candidates for TACE because of massive tumors, bilobar disease, or portal vein thrombosis (PVT). SIRT is also the treatment of choice for patients who are slightly above the criteria for curative treatments and who require tumor down-staging [6]. In recent years, SIRT with Y-90 microspheres has been proposed as an alternative to TACE. Several reports have shown that compared with TACE, SIRT is safe, with a lower rate of embolic complications such as fever and abdominal pain. SIRT is a recommended treatment for advanced HCC patients with PVT and has been shown to be efficacious, with comparable tumor response and survival rates to those of TACE [6]. This study evaluated the efficacy and safety of SIRT for unresectable HCC patients and analyzed prognostic factors affecting overall survival (OS) in this group of patients.

## Patients and methods

### Patients

We enrolled 97 unresectable HCC patients who underwent SIRT with Y-90 microspheres (SIR-spheres, Sirtex Medical, Sydney, Australia) at a single institution between October 2013 and March 2019. Starting in October 2013, all consecutive patients with HCC who were unsuitable for radical treatments (surgery, liver transplantation, or percutaneous ablation) or chemoembolization as a result of the presence of PVT or extensive tumor burden were assessed for SIRT. The inclusion criteria were patients diagnosed with HCC not amenable to curative surgical resection; at least 18 years of age; Eastern Cooperative Oncology Group (ECOG) performance status 0–2; Child–Pugh score ≤ 7; life expectancy > 3 months; without extrahepatic tumor burden; arteriovenous lung shunting < 20% in 99mTc-macroaggregated albumin (MAA); and adequate hematology (granulocyte count ≥ 1.5 × 10^9^/L, platelets ≥ 50 × 10^9^/L), renal function (creatinine level ≤ 176.7 µmol/L), hepatic function (bilirubin level ≤ 2 mg/dL), and transaminase levels < 5 times the institutional upper limit of the normal range. All patients provided informed consent. The exclusion criteria were infiltrative tumor type; tumor volume > 75% of the target liver volume or tumor nodules too numerous to count; tumor volume > 50% combined with an albumin level < 3.0 mg/dL; ascites or clinical liver failure; pre-assessment angiogram showing abnormal vascular anatomy that would result in significant reflux of hepatic arterial blood to the stomach, pancreas, or bowel; disseminated extrahepatic malignant disease; prior external beam radiotherapy involving the liver; female patients who were pregnant or lactating; and severe comorbidities.

The study protocol was performed according to the principles of the 1975 Declaration of Helsinki and the study was approved by the Institutional Review Board of 108 Military Central Hospital (No;3761/HDDD). Informed consent was obtained from all individual participants included in the study.

## Methods

The study was designed as a retrospective investigation. The radioembolization agent was Y-90 Resin Microspheres (SIR-Spheres®, Sirtex Medical Limited, Sydney, Australia) made of 20–60-μm-sized resin or polymer beads with a high-energy, beta-emitting isotope without primary gamma emission [7].

Y-90 resin SIRT protocol: patients underwent comprehensive pretreatment evaluations comprising medical history, physical examination, and laboratory and imaging workup to confirm treatment eligibility. Diagnostic angiography accompanied by 99mTc-MAA scintigraphy was then performed to identify vascular anatomy, HCC feeding vessels, aberrant vessels and extrahepatic collateral vessels feeding extrahepatic organs (especially the gastrointestinal tract), and the presence of intrahepatic or intratumoral arterioportal shunting; and, in case of PVT, the presence of bypassing blood flow through collateral vessels. Aberrant hepatic vessels and extrahepatic collaterals were coil-embolized to prevent the inadvertent misplacement of 90Y resin microspheres into the gastrointestinal tract or pancreas. 99mTc-MAA particles were then injected with the delivery catheter in the intended position for 90Y resin microsphere infusion. Single-photon emission CT (SPECT) images were acquired to evaluate the 3D distributions of the microspheres inside the tumor and surrounding liver. The scintigraphy studies were usually performed within 1 h after 99mTc-MAA injection to assess pulmonary shunt and any unintended flow to other extrahepatic organs. Any patient who exhibited an intense mismatch between intrahepatic 99mTc-MAA distribution and liver lesions as viewed on fusion imaging with SPECT and CT was ineligible for treatment. The doses of resin microsphere treatments were calculated to maximize the therapeutic activity to tumorous tissue and minimize exposure of nontumoral parenchyma and lung tissue. Within 1 week after MAA angiography, SIR-Spheres® were administered following the same route used for MAA. Treatment protocol was approved institutionally.

Data collection and follow-up: the following data were collected at baseline: age, sex, presence of cirrhosis, PVT (segmental, branch, or main) and liver disease involvement (left lobe, right lobe, both lobes), extent of hepatic involvement (number and size of nodules) via CT, laboratory parameters including complete blood cell count, liver function tests, serum creatinine, Anti-HCV antibodies, hepatitis B surface antigen, ECOG, BCLC stages and Child–Pugh scores. Adverse events were classified for according to Common Terminology Criteria for Adverse Events, version 3.0. Tumor response following SIRT was assessed at 3 and 6 months by CT scan. In the evaluation of tumor response, the parenchymal and intravascular components of disease were assessed. With regards to changes in size of target lesions assessed according to mRECIST (8) in those patients with measurable disease, and the development of new lesions was assessed in all patients. If patients had PVT, changes in the extent of PVT were evaluated and classified as partial response (clearance or regression of tumor thrombus into a more distal portal vein segment), stable disease (no changes), or progressive disease (progression of tumor thrombus into a more proximal portal vein segment). Eventually, overall response was established as progressive disease whenever a patient had tumor progression in any of the three individual parameters (measurable target lesions, new lesions, or tumor thrombus) and controlled disease in any other case. Survival was determined from the time of treatment until death. Patients lost to follow-up were censored at the date of their last known visit.

### Statistical analysis

Descriptive statistics for nominal, ordinal, and continuous variables, including frequency, median, and average, were used as appropriate. Proportions were compared using chi-squared tests with continuity correction or Fisher’s exact tests, as appropriate. Survival was calculated from the day of treatment using the Kaplan–Meier method, and subgroup comparisons were performed using log-rank tests. We assessed the predictors of survival using univariate and multivariate Cox proportional hazard regression analyses. To assess the variation in each liver function test over the previous follow-up, we used one-way analysis of variance. The analysis was performed using IBM SPSS Statistics for Windows, version 20.0 (SPSS GmbH, Munich, Germany). Significant differences were defined as p < 0.05.

## Results

### Patient characteristics at baseline

Overall, 97 patients with HCC underwent treatment with Y-90 microspheres with the median follow-up period for all patients after SIRT was 16.4 months (range 1.8–62 months). Ten patients were lost to follow-up within 3 months after TARE. Twenty-three patients who had non-measurable parenchymal target disease (diffuse or massive tumors) did not perform computerized tomography at 6 months after TARE. It was impossible to evaluate for tumor response.

The mean age was 60.4 ± 12.3 (range 25–89) years; 90 patients were male (92.8%) and about half of them were positive for hepatitis B. Most patients had ECOG 1 (73.2%); 19.6% received prior treatments, including resection, radiofrequency ablation, percutaneous ethanol injection therapy, and TACE. Ninety-one patients (93.8%) had a Child–Pugh score of A. Barcelona Clinic Liver Cancer (BCLC) stage C (n = 59, 60.8%) was more common than BCLC B (n = 38, 39.2% and 61 patients (62.9%) had portal vein invasion. The mean tumor size was 10.2 ± 3.5 cm. The disease was limited to one lobe in 81 patients, and 16 patients had bilobar disease (Table 1). The mean dose of Y-90 was 1.6 ± 0.6 GBq, with an estimated 5.2 ± 3.6% being shunted to the lungs.

**Table 1:** Baseline characteristics

Variables	N = 97 (%)
Age, years	
< 65	61 (62.9)
≥ 65	36 (37.1)
Sex	
Male	90 (92.8)
Female	7 (7.2)
Etiology	
Hepatitis B	54 (55.7)
Hepatitis C	4 (4.1)
Alcohol	3 (3.1)
Unknown	36 (37.1)
Performance status	
0	14 (14.4)
1	71 (73.2)
2	12 (12.4)
Prior treatment	
None	78 (80.4)
Resection	5 (5.2)
Radiofrequency ablation	3 (3.1)
Percutaneous ethanol injection therapy	2 (2.1)
Transarterial chemoembolization	9 (9.3)
Method of diagnosis	
Liver biopsy	59 (60.8)
Imaging	38 (39.2)
Pathology	
Well-differentiated	10 (10.3)
Moderate differentiated	38 (39.2)
Poorly differentiated	3 (3.1)
No defined	8 (8.2)
Cirrhosis on imaging	
Present	94 (96.9)
Absent	3 (3.1)
Tumor distribution	
Unilobar, right	74 (76.3)
Unilobar, left	7 (7.2)
Bilobar	16 (16.5)
Portal vein invasion	
None	36 (37.1)
Below subsegmental	1 (1.0)
Subsegmental	28 (28.9)
Lobar	25 (25.8)
Main	7 (7.2)
Index lesion size (cm)	
< 5	6 (6.2)
5–10	40 (41.2)
> 10	51 (52.6)
Tumor burden* (%)	
< 25	44 (45.4)
25–50	38 (39.2)
> 50	9 (9.3)
No defined	6 (6.2)
Alpha-fetoprotein	
< 100	45 (46.4)
≥ 100	52 (53.6)
Total bilirubin (mg/dL)	
< 2	93 (95.9)
2–-3	4 (4.1)
> 3	0 (0)
Albumin (mg/dL)	
> 3.5	83 (85.6)
2.8–3.5	12 (12.4)
< 2.8	2 (2.1)
Child–Pugh class	
A	91 (93.8)
B	6 (6.2)
C	0 (0)
BCLC^†^ stage	
A	0 (0)
B	38 (39.2)
C	59 (60.8)
D	0 (0)

*Tumor burden, Tumor volume/liver volume

^†^BCLC, Barcelona Clinic Liver Cancer staging system

### Treatment response

Among all patients, 64 were followed up beyond 6 months after radioembolization therapy and underwent follow-up CT based on which tumor response was assessed according to the RECIST guidelines. Complete response (CR) and partial response (PR) to treatment were observed in 12 (18.8%) and 23 patients (35.8%), respectively, while 8 (12.5%) and 21 (32.8%) patients, respectively, had stable disease (SD) and progressive disease (PD). The tumor diameter decreased from a mean of 10.2 ± 3.5 cm at baseline to 6.9 ± 3.3 cm at 6 months after treatment (Table 2).

**Table 2:** Tumor response based on mRECIST guidelines

Time point	Lesion diameter (cm), mean ± standard deviation	Number with available data (N = 97)	mRECIST*, n (%)			
			CR	PR	SD	PD
Baseline	10.2 ± 3.5	97	–	–	–	–
3 months	8.2 ± 3.5	87	10 (11.5)	42 (48.3)	19 (21.8)	16 (18.4)
6 months	6.9 ± 3.3	64	12 (18.8)	23 (35.9)	8 (12.5)	21 (32.8)

*mRECIST, Response Evaluation Criteria In Solid Tumors; CR, complete response; PR, partial response; SD, stable disease; PD, progressive disease

**Fig. 1:**
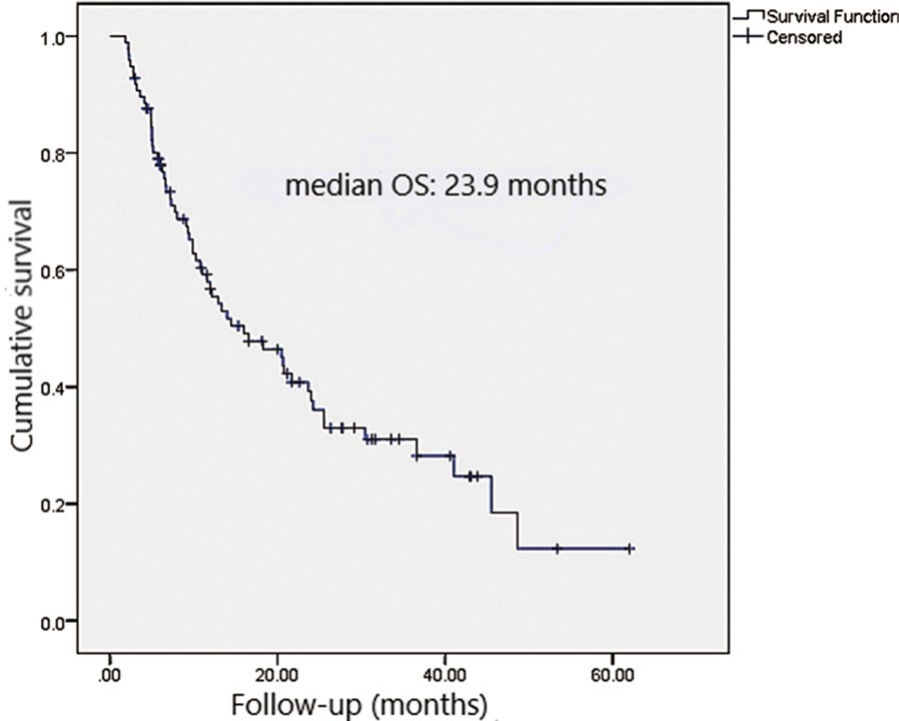
Kaplan–Meier survival curves. (A) Median overall survival was 23.9 months. The median survival after SIRT was 23.9 months with 6-, 12-, 24-, 36-, and 48-month survival of 77.9%, 56.7%, 39.2%, 31%, and 18.5%, respectively

The median survival after SIRT was 23.9 months, with the 6-, 12-, 24-, 36- and 48-month survival rates being 77.9%, 56.7%, 39.2%, 31%, and 18.5%, respectively (Fig. 1). Two patients had SD, with a survival of 48 months. 61 patients died 1 – 48 months after SIRT. In 53 of the 61 patients who died, tumor progression was the suspected cause of death. Sites of progression included target lesions (n = 20), new lesions (n = 12), tumor thrombus (n = 9) and extrahepatic metastases (n = 12). Among the 64 patients who reached 6-month follow-up and were evaluable for response, 21 (32.8%) had progressive disease. Sites of progression included new lesions (n = 19) and tumor thrombus (n = 2).

Overall, univariate analysis of OS showed significant interrelations between age, tumor size, tumor burden, tumor distribution, BCLC stage, and radiological response to treatment. Survival did not differ significantly in subgroups of patients according to sex, ECOG score, prior treatment, portal vein invasion, alpha-fetoprotein (AFP) level, and Child–Pugh score (Table 3).

**Table 3:** Significant predictors of survival

Variables		Univariate analysis		Multivariate analysis	
		HR (95% CI)^*^	*p *Value	HR (95% CI)	*p *Value
Age	< 65	1.97 (1.12–3.46)	0.017		
	≥ 65	1			
Sex	Male	1.25 (0.45–3.49)	0.661		
	Female	1			
ECOG	0, 1	1			
	2	1.39 (0.18–6.67)	0.732		
Prior treatment	No	0.96 (0.51–1.81)	0.893		
	Yes	1			
Tumor distribution	Unilobar	0.87 (0.43–0.98)	0.044	0.10 (0.02–0.75)	0.012
	Bilobar	1			
Portal vein invasion	No	0.61 (0.35–1.06)	0.083		
	Yes	1			
Index lesion size (cm)	≤ 10	0.39 (0.22–0.67)	0.001	0.92 (0.26–3.21)	0.672
	> 10	1			
Tumor burden^†^ (%)	≤ 50	0.18 (0.07–0.43)	0.001	0.94 (0.58–9.52)	0.733
	50–75	1			
Alpha-fetoprotein	< 100	0.63 (0.38–1.05)	0.072		
	≥ 100	1			
Child–Pugh class	Child A	1			
	Child B	1.27 (0.40–4.06)	0.691		
BCLC stage^‡^	BCLC B	0.48 (0.26–0.89)	0.018	0.43 (0.12–1.15)	0.317
	BCLC C	1			
Radiologic response (mRECIST^§^)	CR/PR	0.14 (0.06–0.32)	0.001	0.09 (0.03–0.29)	0.001
	SD/PD	1			

*HR, hazard ratio; CI, Confidential Index

^†^Tumor burden, Tumor volume/liver volume

^‡^BCLC, Barcelona Clinic Liver Cancer staging system

^§^mRECIST, Response Evaluation Criteria In Solid Tumors; CR, complete response; PR, partial response; SD, stable disease; PD, progressive disease

**Fig. 2:**
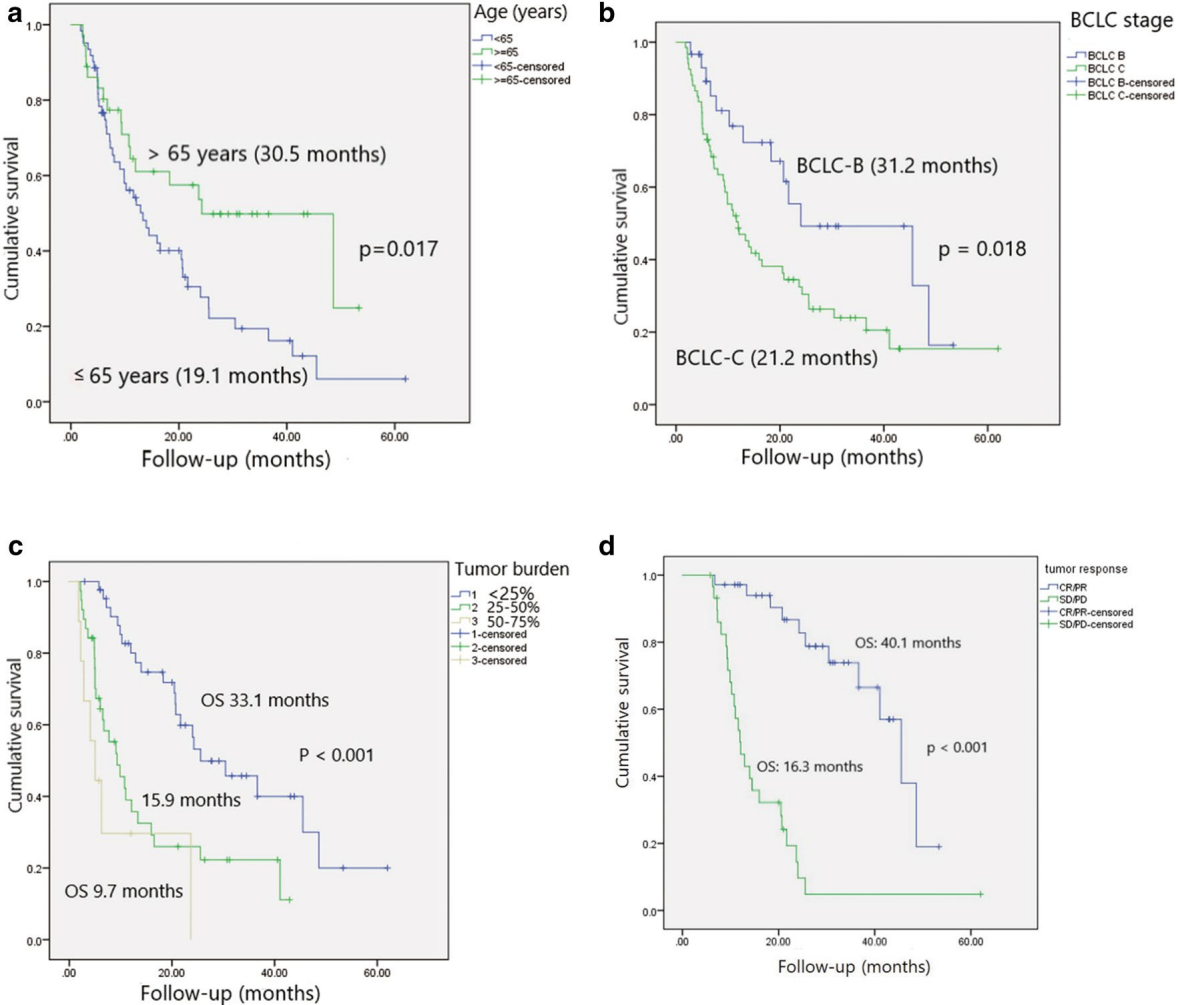
Kaplan–Meier curves demonstrating survival based on age, BCLC stage, tumor burden, and tumor response. **a** The median overall survival is significantly better in patients aged above 65 years than in those aged below 65 years (30.5 vs. 19.1 months; *p* = 0.017). **b** The median overall survival was significantly better in patients with BCLC stage B than in those with BCLC stage C (31.2 vs. 21.2 months; *p* = 0.018). **c** The median overall survival was significantly better in patients with tumor burden under 25% than in those with tumor burden 25–50% and above 50% (33.1, vs. 15.9 and 9.7 months; p < 0.001). **d** The median overall survival was significantly better in patients with objective tumor response (CR/PR) than in those with SD/PD (40.1 vs. 16.3 months; p < 0.001)

Significant differences were noted in terms of survival during 5 years follow-up between Kaplan–Meier survival curves of the two groups according to age, BCLC stage, tumor burden and objective tumor response (Fig. 2a–d), using the log-rank test (*p* value = 0.017, 0.018, < 0.001, and < 0.001, respectively).

In multivariate analysis, the tumor distribution (hazard ratio [HR] for unilobar vs. bilobar; 0.10, confidential interval; 0.02–0.75, *p* = 0.012) and best overall mRECIST response (HR for CR/PR vs SD/PD; 0.09, confidential interval; 0.03–0.29, *p* = 0.001) were independent predictors of OS (Table 3).

### Clinical toxicity

In total, 38 (39.2%) patients developed clinical toxicity after treatment, which included abdominal pain (n = 25, 25.8%), fatigue (n = 19, 19.6%), fever (n = 6, 6.2%), and nausea/vomiting (n = 6, 6.2%). These complications were mild and resolved without active intervention. No patient died because of the treatment.

During follow-up, one patient experienced gastroduodenal ulceration, one experienced pneumonitis, and one experienced gastrointestinal (GI) bleeding 3 months after SIRT. Two patients developed GI bleeding after 6 months.

## Discussion

For many HCC patients, curative surgical therapies (resection or transplantation) are not an option owing to tumor bulk or the presence of metastatic disease. Locoregional therapies such as SIRT with Y-90 are viable alternative methods of reducing tumor burden, prolonging OS, and improving quality of life. Our study demonstrated that SIRT administered to 97 patients with unresectable HCC resulted in a median survival of 23.9 months, with a 3-year survival of 31%. Our encouraging findings are concordant with those reported in earlier series. Salem et al. reported a median survival of 20.5 months and 3-year survival of 25% in 123 patients who underwent SIRT [8]. Mantry et al. reported a median survival of 13.1 months in 111 patients treated with SIRT [9]. In the ENRY study, a prospective European multicenter trial of Y-90 resin SIRT in 325 patients with unresectable HCC, the median OS was 12.8 months (95% confidence interval [CI]: 10.9–15.7) [10]. However, other studies reported longer OS. For instance, Saxena et al. reported a median OS of 27.7 months and 3-year survival of 26% in 45 patients with unresectable HCC [6]. A meta-analysis of 21 published reports showed that the pooled OS was 63% and 27% at 1- and 3-years respectively in intermediate-stage HCC, whereas OS was 37% and 13% at 1- and 3-years respectively in advanced HCC because of the presence of portal vein thrombosis [11].

In patients with advanced HCC, sorafenib has become the standard treatment after the SHARP trial showed a higher median OS for sorafenib (10.7 months) than for placebo (7.9 months) [12]. The SARAH trial demonstrated similar OS between patients treated with SIRT and sorafenib (8.54 vs. 10.58 months), however, a lower rate of serious adverse events was observed in the Y-90 arm (27%) than in the sorafenib arm (> 50%). In addition, the tumor response rate was better in the Y-90 arm (16.5%) than in the sorafenib arm (1.7%) [7]. Collectively, these data support the role of SIRT rather than systemic agents in the management of unresectable HCC, whenever possible.

The current recommended TACE is the standard of care and first-line treatment for intermediate-stage unresectable HCC [13]. The median survival is around 20 months in most series, ranging from 14 to 45 months [14]. Several studies comparing the relative efficacy of SIRT and TACE showed that SIRT is as effective, if not more effective, in inducing a radiological response and improving survival than TACE [15]. Clinically, most of the disadvantages of TACE manifest as pain and post-embolization syndrome, often requiring hospitalization. Moreover, TACE is contraindicated in patients with main PVT because of its profound ischemic effects. Salem et al. compared the results of 122 patients who underwent SIRT with those of 123 patients treated with TACE [8]. Complications, particularly, abdominal pain and liver dysfunction were more common following TACE than following SIRT (p < 0.05). A trend toward higher objective response rates (49 vs. 36%, *p* = 0.1) was observed in patients undergoing SIRT. The time-to-progression was longer following SIRT than following TACE (13.3 vs. 8.4 months, *p* = 0.046); however, the median OS did not differ significantly (20.5 vs. 17.4 months, *p* = 0.2). Particularly, in meta-analysis of 10 published reports, SIRT showed a statistically significant benefit as compared to TACE in terms of higher progression-free survival rate, although SIRT and TACE showed similar overall survival [16]. These studies illustrated better quality of life outcomes in patients undergoing SIRT rather than those undergoing TACE, although more patients presented with advanced disease.

In the univariate analysis, the prognosticators of OS were age, tumor size, liver tumor burden, tumor distribution, BCLC stage, and radiological response to treatment. Patients older than 65 years fared better than their younger counterparts, likely because the proportion BCLC B patients older than 65 years was higher than that in younger patients (55.6% vs. 36.1%, *p* = 0.061). Considering recent reports, radioembolization appears to be a well-tolerated and effective treatment option for elderly patients without concomitant disease [8, 17]. In our study, the median OS was significantly better in patients with BCLC stage B than in those with stage C (31.2 vs. 21.2 months; *p* = 0.018). Salem et al., Ozkan et al., and Sangro et al. also emphasized the significant relationship between BCLC stage and survival [8, 10, 18]. Multivariate analysis in the present study showed that unilobar tumor distribution and tumor response were prognostic factors for OS. This finding was expected and has been reported previously. Mazzaferro et al. showed that radiological response (CR/PR/SD vs. PD) was an independent risk factor for OS (*p* = 0.048) [19].

In this study, the clinical toxicity rate was 39.2%, and all events, including abdominal pain, fatigue, fever, and nausea/vomiting, were self-limiting and resolved without active intervention. No treatment-related mortality was observed, similar to reports from earlier series[6, 8]. During the follow-up, one patient developed dyspnea 3 months after radioembolization, suggesting radiation-induced pneumonitis. One patient presented with gastroduodenal ulceration and one case had variceal esophagus bleeding 3 months after treatment. At 6 weeks after SIRT, two patients developed GI bleeding owing to hepatic failure. Mayer et al. reported about three patients with gastroduodenal ulcers, one with radiation pneumonitis, and one with septicemia [20].

Our study also has some limitations. First, the retrospective nature of this study and the relatively small sample size obviously limits the strength of evidence. Second, the median follow-up period for all patients was too short. In particular, the presence of a high percentage of patients for whom the tumor response could not be evaluated. It is expected to have affected these results. The hazard ratios of the tumor distribution and the best overall mRECIST response (CR/PR) were likely to be overestimated. It is another limitation for the results of this study. Third, we were not able to analyze the severity of non-laboratory adverse events because the description of the adverse events is not properly recorded. Finally, the Kaplan–Meier method is the most commonly used time-to-event analysis. However, since not all the patients died because of cancer, a survival method that considers the competing risk would be more appropriate.

## Conclusions

Our study results add to the growing literature on SIRT for unresectable HCC, demonstrating that SIRT is safe and effective with high objective response rates (CR/PR 54,7%) and median OS of 23.9 months. The unilobar disease before SIRT and tumor response (CR/PR) are significant predictors of survival.

## Data Availability

The datasets generated and analyzed during the current study are available from the corresponding author on reasonable request.

## Abbreviations

BCLC: Barcelona clinic liver cancer
CR: Complete response
CT: Computed tomography
TACE: Transarterial chemoembolization
GI: Gastrointestinal
HCC: Hepatocellular carcinoma
HR: Hazard ratio
MAA: 99MTc-macroaggregated albumin
mRECIST: Modified response evaluation criteria in solid tumor
OS: Overall survival
PD: Progressive disease
PR: Partial response
PVT: Portal vein thrombosis
SD: Stable disease
SIRT: Selective internal radiation therapy
SPECT: Single-photon emission computed tomography
Y-90: Yttrium-90
